# Assessment of Emotional Experience and Emotional Recognition in Complicated Grief

**DOI:** 10.3389/fpsyg.2016.00126

**Published:** 2016-02-12

**Authors:** Manuel Fernández-Alcántara, Francisco Cruz-Quintana, M. N. Pérez-Marfil, Andrés Catena-Martínez, Miguel Pérez-García, Oliver H. Turnbull

**Affiliations:** ^1^Mind, Brain and Behavior Research Center, University of GranadaGranada, Spain; ^2^School of Psychology, Bangor UniversityBangor, UK

**Keywords:** complicated grief, emotion, affective neuroscience, bereavement, emotional assessment, IAPS

## Abstract

There is substantial evidence of bias in the processing of emotion in people with complicated grief (CG). Previous studies have tended to assess the *expression* of emotion in CG, but other aspects of emotion (mainly emotion recognition, and the subjective aspects of emotion) have not been addressed, despite their importance for practicing clinicians. A quasi-experimental design with two matched groups (Complicated Grief, *N* = 24 and Non-Complicated Grief, *N* = 20) was carried out. The Facial Expression of Emotion Test (emotion recognition), a set of pictures from the International Affective Picture System (subjective experience of emotion) and the Symptom Checklist 90 Revised (psychopathology) were employed. The CG group showed lower scores on the dimension of valence for specific conditions on the IAPS, related to the subjective experience of emotion. In addition, they presented higher values of psychopathology. In contrast, statistically significant results were not found for the *recognition* of emotion. In conclusion, from a neuropsychological point of view, the *subjective* aspects of emotion and psychopathology seem central in explaining the experience of those with CG. These results are clinically significant for psychotherapists and psychoanalysts working in the field of grief and loss.

## Introduction

All human beings confront experiences of loss. Our coping mechanisms are shaped by a number of factors, including personality, support from others and a range of social influences (Bonanno et al., 2011; Mancini et al., 2012). Several scientific findings provide evidence that most people experiencing the death of a loved one have sufficient resources to cope with this loss (Bonanno and Kaltman, 2001; Bonanno and Diminich, 2013). However, 10–15% of people have substantial emotional difficulties, and develop prolonged or complicated grief (CG; Prigerson et al., 2008).

The concept of CG has received much recent attention, and was proposed as a diagnostic category for the DSM-V (Bryant, 2013, 2014). Most diagnostic approaches agree that it can be defined as an intense grief reaction, continuing for at least 6–12 months after the death of a loved one, characterized by emotional dysregulation, including symptoms of yearning, anger, guilt, recurrent sadness, rumination, a difficulty accepting the reality of the loss, and a sense of loss of meaning in life.

Several studies have addressed the issue of emotion in this population, typically using tasks requiring processing of death-related stimuli (Martí-García et al., 2016). For example, Maccallum and Bryant (2010) used an emotional Stroop task (with death-related and neutral words) and found that participants with CG responded more slowly to death-related words, by comparison with non-complicated grievers. This result was replicated in older adults using a counting Stroop task (O’Connor and Arizmendi, 2014). Likewise, patients with CG had rapid accessibility of the deceased’s name, in both control and threathening conditions (Mancini and Bonanno, 2012), and showed an attentional bias toward happy and sad faces when completing an emotional dot-probe paradigm (Bullock and Bonanno, 2013). Similarly, complicated grievers also seem to have problems in emotion regulation, for example when asked to enhance and suppress their emotional expression (Gupta and Bonanno, 2011), as well as less variability and lower expression of emotion in a range of different contexts (Diminich and Bonanno, 2014).

On the other hand, a number of studies have addressed emotion from a *subjective* perspective in CG. For example, cross-sectional studies have identified relationships between CG and a wide range of symptoms of psychopathology, including major depression, post-traumatic stress disorder (PTSD) and anxiety (Simon et al., 2007; Golden and Dalgleish, 2010; Pini et al., 2012; Marques et al., 2013). The same pattern was found with other emotional variables such as separation anxiety (Boelen, 2013) or experiential avoidance (Boelen et al., 2010). These results suggest that CG is associated with a general psychopathological profile of more powerful symptoms, that could, for example, be related to the high rates of medication in this population (Durà-Vilà et al., 2013; Bandini, 2015; García and Landa, 2015).

However, these findings on the nature of CG have yet to extend to the affective neuroscience paradigm (Panksepp, 1998), which has had such a critical role in the development of the current neuropsychoanalytic approach (Solms and Turnbull, 2002). For example, in a review that analyzes the role of PANIC (separation/loss) and SEEKING systems in depression and addiction, little attention is dedicated to the distinction between depression and CG (Zellner et al., 2011). However, recent neuroimaging studies show that the brain circuits activated in patients with CG when facing biographical and death-related emotional stimuli *are* distinguishable from those activated in depressive patients. Notably, they were associated with areas linked to the SEEKING system, where the symptoms of yearning for the deceased were related to activation on the nucleus accumbens (O’Connor et al., 2008). In addition, different patterns linked to emotion regulation have also been reported, involving less activation in the anterior cingulate cortex (ACC) and orbitofrontal cortex for those with CG (Arizmendi et al., 2016).

In sum, there is evidence of alterations and bias on several aspects of emotion in CG, including death-related stimulus processing, emotion regulation, and substantial enhancement of psychopathological symptoms. However, some central aspects of emotional processing on this population remain unclear.

From a neuropsychological point of view, there is a clear distinction between the *perception and expression* of emotions, and the *subjective experience* of emotion (Panksepp, 1998; Adolphs and Damasio, 2000; Damasio et al., 2000; Panksepp and Watt, 2011; Carmona-Perera and Pérez-Garcia, 2012; Salas et al., 2012; Bueso-Izquierdo et al., 2015), which may also relate to the categorical versus the dimensional approach to emotion (Carmona-Perera and Pérez-Garcia, 2012). This distinction seems especially critical for the psychotherapist or psychoanalyst, whose work is focused on the subjective elements of the client’s presentation.

In order to assess the perception and expression of emotions, two classical tasks are widely used: the *Facial Expressions of Emotion Test* (FEEST) for emotional perception (Young et al., 2002) and the *Facial Action Coding System* (FACS) for emotional expression (Ekman and Friesen, 1975). To elicit the subjective experience of emotion, the *International Affective Picture System* (IAPS) has long been employed, through the dimensions of valence, arousal, and dominance (Lang and Bradley, 2010). Finally, the symptom profile can be assessed using self-report measures that cover a wide range of these alterations (Derogatis, 2002).

With the exception of Diminich and Bonanno (2014), which measured the expression of emotion through the FACS, no previous studies have assessed emotional recognition, subjective experience and profile of psychopathology in a single sample of clients with CG and non-CG.

The main objective of the current research was to evaluate emotion recognition, subjective experience and psychopathology in patients diagnosed with CG, by comparison with non-CG. Because of the central role of the *experience* of emotion in psychopathology, and because of the previous research reviewed above, we hypothesize that those suffering from CG will show differences in the subjective experience of emotion, and greater psychopathological symptoms, while there will be no significant differences in emotion recognition.

## Materials and Methods

### Participants

A total of 47 participants took part on the research (see **Table 1**). They were recruited from the Clinical Unit at the Faculty of Psychology in Granada (Spain), the Palliative Care Unit of San Cecilio Clinical Hospital (Granada – Spain), and from two associations supporting grief and bereavement: Alma y Vida (Jaén- Spain) and Talitha (Albacete – Spain). The inclusion criteria were: (a) having experienced the death of a loved one in the last 6 months and (b) being 18 years-old or more. The exclusion criteria were: (a) having writing and/or reading problems, (b) having a previous diagnosis of psychiatric illness, and (c) an extreme score (>75th Percentile) on the variables of depression and/or PTSD. Three participants were excluded due to criterion number three. The final sample had 44 participants (**Table 1**).

**Table 1:** Demographic characteristic of the sample divided by groups.

Variable	Complicated Grief (*N* = 24)	Non-Complicated Grief (*N* = 20)	Test statistic
	Mean or *N* (*SD* or %)	Mean or *N* (*SD* or %)	*t* or χ^2^
Age	41.42 (13.87)	45.40 (11)	ns
Months since death	28.17 (18.8)	35.30 (34.99)	ns
Years of education	13.38 (3.94)	13.60 (3.69)	ns
Gender			ns
Female	17 (70.84%)	15 (75%)	
Male	7 (29.16%)	5 (25%)	
Relationship with the deceased			ns
Partner	3 (12.5%)	4 (20%)	
Child	9 (37.5%)	5 (25%)	
Parent	7 (29.1%)	10 (50%)	
Sibling	1 (4.2%)	1 (5%)	
Others	4 (16.7%)	0 (0%)	
Inventory of Complicated Grief	39.62 (9.78)	17.45 (5.21)	*p* < 0.001
Beck Depression Inventory	13.83 (7.46)	6.55 (5.89)	*p* = 0.001
PTSD Global Assessment (EGEP)			
Number of symptoms of re-experience	3.43 (1.16)	2.26 (1.48)	*p* = 0.007
Intensity of symptoms of re-experience	8.82 (5.86)	4.33 (4.26)	*p* = 0.003
Number of symptoms of avoidance-affective numbness	3.65 (2.08)	1.68 (1.70)	*p* = 0.002
Intensity of symptoms of avoidance-affective numbness	8.14 (5.86)	2.67 (3.36)	*p* = 0.001
Number of symptoms of hyper-arousal	3.13 (1.57)	1.89 (1.24)	*p* = 0.008
Intensity of symptoms of hyper-arousal	6.41 (4.18)	2.72 (2.22)	*p* = 0.002

The Inventory of Complicated Grief was used to divide the sample in two groups (*N* = 24 for the CG group, and *N* = 20 for the non-CG group). A score of 25 or higher was the criterion for CG (Prigerson et al., 1995). Both groups showed no statistical difference in gender, months since death, years of education and the type of relationship with their loved one. However, as expected, they showed differences in their scores on measures of depression, CG, and PTSD (see **Table 1**).

### Instruments

#### Complicated Grief

The Inventory of Complicated Grief (ICG: Prigerson et al., 1995; Spanish version: Limonero- García et al., 2009) is a self-report measure, composed of 19 items that assess the appearance of symptoms related to CG (on a five point Likert scale ranging from 0 to 4), including: yearning (*“I feel myself longing for the person who died”)*, anger *(“I can’t help feeling angry about his/her death”*), shock (*“I feel stunned or dazed over what happened”*), or emptiness (*“I feel that life is empty without the person who died”*). Psychometric studies show high internal consistency (Cronbach’s α = 0.94) and test–retest reliability in its original, as well as in the Spanish version (α = 0.88, Test–retest reliability = 0.81). This instrument provides a single score for the intensity of grief symptomatology, and has been widely used as a valid measure to distinguish between CG and Non-CG, in a number of cross-sectional studies (Kersting et al., 2011; Eisma et al., 2013).

#### Depression

The Beck Depression Inventory (BDI: Beck et al., 1961; Spanish version: Sanz and Vázquez, 1998) is a self-report measure that assesses depressive symptomatology, including feelings of sadness, being discouraged about the future, or guilt. It is widely used in clinical and experimental research, and has been validated for a Spanish population. Previous studies suggest an adequate reliability (α = 0.89), as well as satisfactory evidences of validity.

#### Post-Traumatic Stress Disorder

The Global Assessment of Post-traumatic Stress (EGEP: Crespo and Gómez, 2012) is a self-report measure used to assess PTSD. It is comprised of 62 items that give a measure of the number of traumatic events experienced by the participant, relating these to symptoms of PTSD. The scale assesses symptoms of re-experience (*“I have unpleasant and repetitive dreams about the event”*), avoidance and affective numbness (*“I try to avoid thoughts, feelings or conversations related to the event”*) and hyperactivation (*“I’m in a constant state of alertness or vigilance”*). The instrument was created using a Spanish sample, and data suggest acceptable psychometric properties (α ranging from 0.73 to 0.86 for the different subscales).

#### Emotional Recognition

The FEEST was used (Young et al., 2002). This task is comprised of 60 images of faces from the Ekman and Friesen series of Pictures of Facial Affect (Ekman and Friesen, 1975) that show six types of emotions: anger, fear, sadness, happiness, disgust, and surprise. The CD-ROM version was employed, which uses photographs of the faces of a total of 10 people. An example of each emotion was shown to participants before the beginning of the task. Each face was randomly presented for a maximum interval of 5 s, followed by a blank screen. A total score of correct responses and a single score for each emotion were obtained.

#### Subjective Emotional Experience

Twenty-five images from the (IAPS: Lang et al., 2005; Spanish Adaptation: Moltó et al., 1999; Vila et al., 2001) were selected, depending on their valence and arousal normative values, to obtain an adequate representation of the different emotional space (see **Table 2**). A total of five conditions of pictures were selected, based on a modification of the Clinical Evaluation of Emotional Response Instrument (ICERE- Instrumento Clínico de Evaluación de la Respuesta Emocional: Aguilar de Arcos et al., 2005). The stimuli selected have been previously employed in research involving end-of-life processes (Montoya-Juárez, 2011).

**Table 2:** Images used from the IAPS in the five conditions and mean values of valence and arousal for each category.

Condition	Valence	Arousal	Dominance	IAPS picture number	Mean valence	Mean arousal
1 – N	Neutral	Calm	Neutral	2190;7224;7233;7235;7950	5.02	2.60
2 – UNA	Unpleasant	Neutral	Low	2280;2491;2520;2715;3180	3.45	4.18
3 – UA	Unpleasant	Arousing	Low	1050;2688;2691;2900;3350	2.34	7.01
4 – PNA	Pleasant	Neutral	High	2360;2550;5000;5551;7472	7.46	3.37
5 – PA	Pleasant	Arousing	High	5621;8040;8260;8496;8502	7.18	6.34

To assess the emotional experience to these pictures, the Self-Assessment Manikin (SAM) was used (Bradley and Lang, 2000), in its pencil-and-paper version. It is an useful instrument to characterize the subjective experience of emotion associated with the processing of most stimuli. This instrument uses pictorial scales to assess three emotional dimensions: valence, arousal, and dominance. Each scale of the SAM consists of a set of five humanoid figures; with nine possible levels of intensity (each figure and interval between the figures represents a level of intensity).

#### Emotional Psychopathology

The Symptom Checklist-90 Revised (SCL-90-R: Derogatis, 2002) is a brief, multifaceted self-reporting questionnaire, designed to explore a wide range of psychopathological symptoms. The SCL-90-R provides a measure of three related aspects of psychopathology: global, dimensional, and discrete. It also targets three different levels of symptoms: three global indices, nine subscales with indices of primary mental health symptoms, and one index of discrete symptoms.

It measures features of somatization (*“Headaches”*), obsessive-compulsive symptoms (*“Unwanted thoughts or ideas that won’t leave your head”*), interpersonal sensitivity (*“Feeling critical of others”*), depression (*“Feeling lonely”)*, anxiety (*“Nervousness or shakiness inside”*), hostility (*“Feeling easily annoyed or irritated”*), phobic anxiety (*“Feeling afraid in open spaces or on the street”*), paranoid ideation (*“Feeling others are to blame for most of your troubles”*), and psychoticism (*“The idea that someone can control your thoughts”*). The SCL-90-R is validated in Spanish population and has normative data (Derogatis, 2002). The reliability in the Spanish sample varied from α = 0.81 to α = 0.87 on the different subscales.

### Procedure

Participants that accepted to take part in the study, and met inclusion criteria, completed a large number of neuropsychological tasks and self-report measures. Participation in the study was voluntary, and participants did not receive financial or other compensation. They were contacted through the database of patients from San Cecilio Clinical Hospital, and Associations Talitha and Alma y Vida. Each participant performed a total of three sessions, each of them of one and half hours duration. In the first session they completed a sheet with all the self-report questionnaires. During the second session they completed the IAPS and the FEEST, which were presented in the same order to all participants. The last session was used to assess other neuropsychological variables. All sessions were performed in a quiet location, administered by the same researcher, and under homogeneous conditions.

The research was approved by the Ethical Committee of Human Research at the University of Granada (Spain). All participants were informed of the objectives of the study and were given an information sheet. Written informed consent was collected from all participants before the start of the study.

### Data Analysis

First, mean and standard deviation were calculated for each group (CG versus Non-CG), based on the total scores of each task. Second, to test if there were significant differences between groups, *t*-tests for independent samples and bifactorial MANOVAS were carried out, followed by *post hoc* comparisons when the results were statistically significant. Cohen’s *d* and partial-eta squared values were calculated as a measure of effect size. Holm–Bonferroni correction was used for controlling multiple comparisons and *p*-values were set below 0.02.

## Results

### Emotional Recognition

There were no differences between groups in the total number of emotional faces recognized on the FEEST, *t*(41) = 0.436, *p* = 0.66, *d* = 0.13 or in any of the discrete emotions (anger, surprise, sadness, happiness, disgust, and fear).

### Subjective Emotional Experience

To analyze the different emotional categories from the IAPS, three bifactorial MANOVAS were completed, having group as an independent variable (CG versus Non-CG) and the scores on each dimension (valence, arousal and dominance) as dependent variables. There were statistically significant differences for the valence dimension, *F*(5,38) = 3.27, *p* = 0.015, $\eta_{p}^{2}$ = 0.301, Wilks’ Lambda = 0.699, and marginally significant differences for the arousal dimension, *F*(5,38) = 2.39, *p* = 0.055, $\eta_{p}^{2}$ = 0.240, Wilks’ Lambda = 0.760. Scores on dominance were not different between groups, *F*(5,38) = 1.26, *p* = 0.30, $\eta_{p}^{2}$ = 0.142, Wilks’ Lambda = 0.858.

**FIGURE 1:**
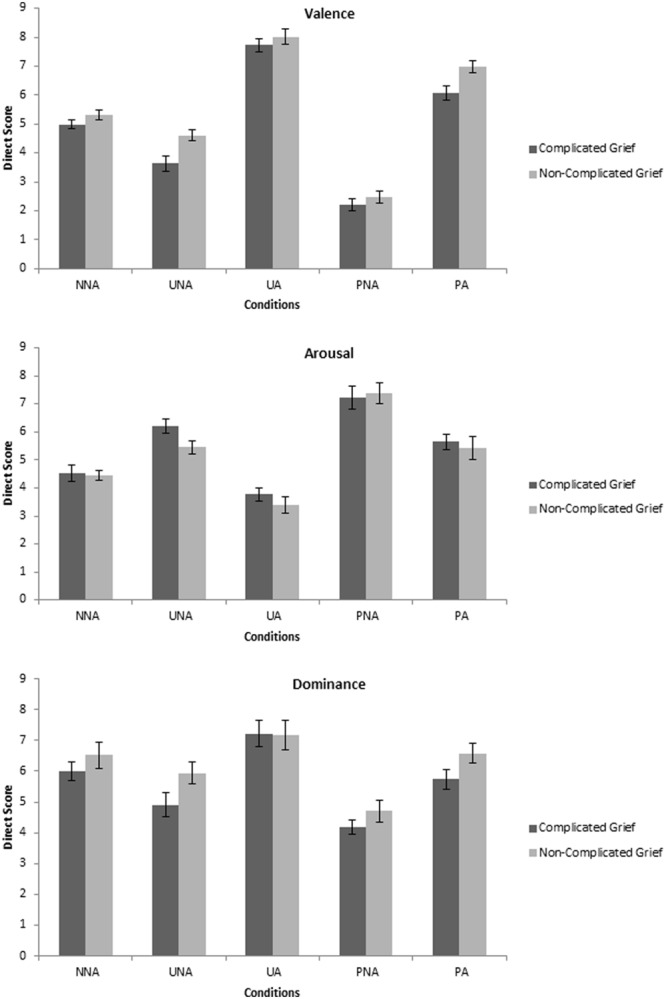
**Means of each dimension of the IAPS depending on the pictures conditions**.

*Post hoc* comparisons showed statistically significant differences for the valence of the unpleasant non-arousing pictures (UNAs), *t*(42) = 2.75, *p* = 0.009, *d* = 0.86 and the pleasant arousing pictures (PA), *t*(42) = 2.70, *p* = 0.01, *d* = 0.84. Results indicated that the CG group perceived the images of these categories as more unpleasant than the Non-CG group (see **Figure 1**).

### Emotional Psychopathology

**FIGURE 2:**
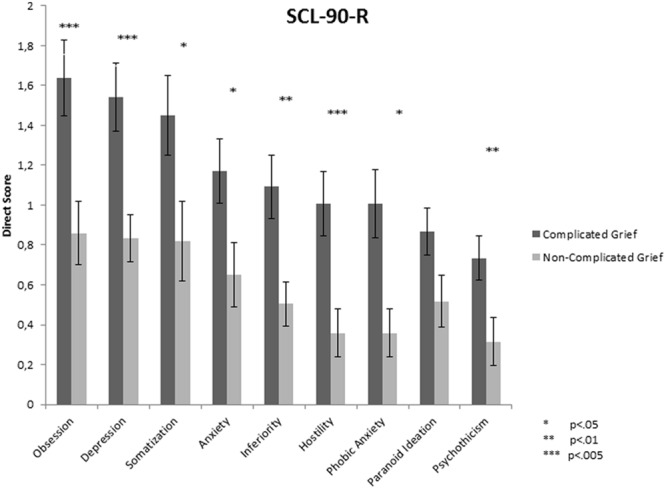
**Means of each dimension of the SCL-90-R (Direct Score) for each group**.

The three main dimensions of emotional psychopathology assessed through the SCL-90-R were the Global Severity Index (GSI), the Positive Symptom Total (PST), and the Positive Symptom Distress Index (PSDI). The GSI values were statistically significant [*t*(42) = 3.2, *p* = 0.003, *d* = 1], as well as the PST [*t*(42) = 3.22, *p* = 0.002, *d* = 1] and the PSDI [*t*(42) = 2.03, *p* = 0.049, *d* = 0.62]. Participants from the CG group showed higher scores on the three indices of global psychopathology. *Post hoc* analysis showed that scores on eight of the nine sub-scales were significantly different between groups, indicating very substantial number of emotional symptoms for those with CG. Five of nine dimensions (obsession, depression, inferiority, hostility, and psychoticism) had *p* values below 0.02 (see **Figure 2**).

Finally, levels of PTSD are presented on **Table 1**. Those with CG had a high number and intensity of symptoms of PTSD, on the dimensions of re-experience (*d* = 1.02), avoidance-affective numbness (*d* = 1.04), and hyper-arousal (*d* = 1.1). A total of 12 participants (50%) from the CG group fulfilled the criteria for PTSD, by comparison with only one participant (5%) from the non-CG group [χ^2^(1) = 10.71, *p* = 0.001].

## Discussion

Research in CG has been wide ranging: from the interference produced by a death-related stimulus, through issues of emotional flexibility and regulation, and also involving the facial expression of emotions. Nevertheless, as far as we know, the present study is the first to study emotion in CG from a neuropsychological perspective using a broad *set* of tasks: to address emotional recognition, the subjective experience of emotion, and psychopathology in the same sample. The results seem to indicate that subjective experience of emotion and emotional psychopathology differs between those with and without CG, while recognition of emotion did not show variation between groups.

### Emotional Recognition

Regarding the recognition of emotion, none of the six Ekman emotion categories differed significantly between groups. Previous studies have reported face recognition as useful in identifying psychopathologies such as depression (Kohler et al., 2011; Jaworska et al., 2012) or PTSD (Felmingham et al., 2003). However, the present results suggest that face recognition tasks in general may be of limited use in identifying patients with CG. Nevertheless, some tasks have produced significant effects using emotional faces. For example, studies using a photograph of the deceased relative triggered loss-reaction responses in those with CG (O’Connor et al., 2008; Eisma et al., 2014). Presumably this is because they are related to the *internal* emotional experience of participants (Salas et al., 2012) and perhaps because none of these experiments directly asked about the *knowledge* of the emotions that were displayed (Adolphs and Damasio, 2000). Also, although it seems that the facial expression of emotion may be altered in CG (Diminich and Bonanno, 2014), our results indicate that accurate recognition of emotions might not be disrupted.

### Subjective Emotional Experience

The findings were strikingly different for the subjective experience of emotion. Previous studies on CG have used pictures from the IAPS (Gupta and Bonanno, 2011), but the present is the first to systematically use it to measure emotional *experience*, controlling for the range of valence and arousal, and assessing the five classical conditions of the emotional space (Lang and Bradley, 2010). The CG group showed substantially different scores on the dimension of valence on two conditions, which included both pleasant and unpleasant pictures, with medium and large effect sizes. These results may be important for understanding the subjective experience in those with CG. Previous studies have reported difficulties in both the enhancement and suppression of emotion in people diagnosed with CG (Gupta and Bonanno, 2011), suggesting a lack of emotional flexibility (Bonanno et al., 2004; Bonanno and Burton, 2013). It is possible that symptoms of CG may lead to an hyperactivation of the aversive motivational system, which make it difficult to regulate both positive and negative emotions (Bradley and Lang, 2000; Lang and Bradley, 2010).

A question that remains unclear is why the differences in valence were so striking on two specific conditions on the IAPS. Following Peter Lang’s theory of emotion, it is interesting to notice that the UNA and PA pictures are opposed categories in the emotional space, both in the American and the Spanish version (Moltó et al., 1999; Vila et al., 2001). In addition, UNA pictures are the most difficult pictures to find; due to the complication in obtaining unpleasant emotional stimulus that do not imply substantial arousal (Moltó et al., 1999, 2013; Lang and Bradley, 2010). Future studies might employ a large number of pictures from each condition, in order to replicate current findings and establish their implications.

The content of the pictures may also have an important role. It is interesting to note that the ratings differed on pictures that were related to sadness and sickness (UNA pictures) and to joyful and arousing situations (PA pictures). We could argue that these two conditions were both linked to loss-related and to restoration-related feelings, as suggested by the Dual Process Model of Coping with Bereavement (Stroebe and Schut, 2010). From this point of view, CG is characterized by a difficulty in oscillating between the loss-oriented and restoration-oriented responses. It is possible that the two conditions on the IAPS reflected this distinction, between loss and restoration affective stimuli (Stroebe and Schut, 2010; Fasse and Zech, 2015). Again, further research is needed to test this hypothesis, using distinct pictures for each category.

### CG and Affective Neuroscience

The differences in subjective emotional experience reported by those with CG may be linked to data from neuroimaging and affective neuroscience. Firstly, the importance of the subjective clearly emphasizes the critical role of the *felt* aspects of emotion (Solms and Turnbull, 2002; Panksepp, 2010). From this point of view, the unpleasant experience of both UNA and PA pictures may be an indication of a disturbance on the level of affect or feelings (Panksepp, 2010). In line with this hypothesis, a wide range of subcortical structures (associated by Panksepp with primary-process networks) have a critical role on the experience of CG. When participants with and without CG were compared (on an emotional task that used stimuli related and unrelated to the deceased) the nucleus accumbens was the critical brain structure that differentiates between groups (O’Connor et al., 2008). Other emotional areas, such as the amygdala or the orbitofrontal cortex, also involved in the evaluation of the reward, have also shown increased activation on those with CG (O’Connor and Arizmendi, 2015).

Secondly, some of the previously discussed brain areas are an integral part of the dopaminergic reward system, which has been suggested to be of central importance for understanding CG. The emotional experience of loss-related stimuli seems to hyper-activate this wanting or SEEKING system (O’Connor et al., 2008; Panksepp and Watt, 2011; Zellner et al., 2011). This pattern is distinct from the one presented in depression, which is characterized by an activation of the PANIC/GRIEF system (mediating separation-distress) and *decreased* activation on the SEEKING system – producing a decrease in arousal, and dysphoric symptoms (Panksepp, 2010; Zellner et al., 2011). Many clinicians will be unaware of this important behavioral distinction (and its neural basis), but the finding may well have implications for the treatment of CG as compared to other forms of depression – from both a psychotherapeutic and a pharmacotheraputic perspective.

Future researches, including neuroimaging studies, are clearly needed to assess the specific role of CG symptoms on these emotional networks, as well as any similarities and differences from depression. One possible way of addressing this objective may be to compare the neural activation of participants diagnosed with only with CG, versus those that also fulfill the criteria of clinical depression. This kind of research may clarify the role of SEEKING and PANIC/GRIEF system on grief.

### Psychopathology and Symptom Profile

Although previous studies *have* investigated psychopathology in CG (Simon et al., 2011; Boelen, 2013) the current research sought to identify the general profile of symptomatology that characterized this population. Emotion-related psychopathology was assessed through the SCL-90-R. Participants with CG showed higher scores on *all* subscales, in line with previous research (Simon et al., 2007; Golden and Dalgleish, 2010; Boelen, 2013). These results suggest, perhaps unsurprisingly, that people with CG are at greater risk of a range of emotional problems. In addition, their symptom profile may thus be easily confounded not only with well-known psychopathologies such as depression, anxiety or PTSD (Bryant, 2014), but also with symptoms that represent obsessive behavior, feelings of inferiority, outbursts of hostility, or even frankly psychotic features.

A consequence of this complex symptom profile is that those with CG may be inappropriately diagnosed and treated, perhaps to control the intense emotions in the early stages of bereavement (García et al., 2013; García and Landa, 2015), leading to later difficulties with grief resolution. This is an under-investigated topic, though in one qualitative study of bereaved mothers, all showed remorse that their medication schedule did not allow them to fully experience loss-related feelings (Bosquet-del Moral et al., 2012). In addition, this misidentification may lead to over-treatment and excessive diagnosis of both depression and CG, due to the new diagnostic criteria for depression of the DSM-5 (Bryant, 2013; Bandini, 2015).

### Clinical Implications

Data from the present study, supporting the key role of *subjective* emotional experience in CG, will be of special interest for clinicians – clearly suggesting that psychotherapeutic intervention should focus on internally focussed elements of the client’s experience. This is in line with many current clinical approaches, mainly focused on the use of specific emotional techniques to promote elaboration and integration of the loss, developing new coping strategies and interpretations of the inner emotional narrative (Boelen et al., 2006; Mancini et al., 2012; Alves et al., 2014). Indeed, there may be a role for strategies such as holding and supporting negative emotional experiences (Winnicott, 1960), which may help to integrate the intense feelings of yearning, sadness and shock into the biographical self (Maccallum and Bryant, 2013). In addition, because of the differences in the role of dopaminergic (SEEKING) systems, there are also implications for the pharmacotherapy of CG, by comparison with other forms of depression (as discussed above). Clearly, this is an important topic for future research.

### Limitations of the Study

There were both strengths and limitations in the study. The principal limitation was the small sample size, which may not have allowed enough statistical power to detect a difference in emotion conditions. However, studies involving patients with CG usually have a range between 15 and 30 participants (Robinaugh and McNally, 2013; O’Connor and Arizmendi, 2014). In addition, effect sizes (independent of sample size) were reported in the present study, and are of a reasonable magnitude. A second issue relates to gender. There is evidence from previous research that men and women may experience and cope with loss in different ways (Chiu et al., 2011; Kersting et al., 2011), and no previous studies have directly addressed gender differences in the study of emotional processing on CG. Due to the limited sample size of the present study it was not feasible to satisfactorily study gender effects. Finally, current research on the subjective aspects of emotion involves taking into account the specificity of an emotional schema related to the death (Martí-García et al., 2016). Future studies might be aimed at identifying whether these differences in subjective emotional processing are related to the activation of this death-related schema.

## Conclusion

The present study suggests that the subjective experience of emotion seems central for understanding the nature of CG, while emotional perception is relatively unaffected. This symptom profile has to be taken into account to avoid diagnostic errors, and has further clinical implications. These results are also consistent with evidence from affective neuroscience, linking CG with addiction, due to their shared cerebral mechanisms. Further studies, using a larger set of emotional tasks, would be welcome, to replicate and extend the results presented here – as is work to better understand the neural basis of these effects.

## Author Contributions

MF-A, FC-Q, MP-M, MP-G, and AC-M designed the tasks. MF-A, FC-Q, and MP-M collected the data. MF-A, AC-M, MP-G, and OT undertook statistical analysis, and MF-A wrote the first draft of the manuscript. All authors contributed to and have approved the final manuscript.

## Conflict of Interest Statement

The authors declare that the research was conducted in the absence of any commercial or financial relationships that could be construed as a potential conflict of interest.
